# Blink-induced changes in pupil dynamics are consistent and heritable

**DOI:** 10.21203/rs.3.rs-4718613/v1

**Published:** 2024-08-06

**Authors:** Şükrü Barış Demiral, Nora D. Volkow

**Affiliations:** National Institutes of Health; National Institutes of Health

**Keywords:** Blink, pupil, drowsiness, reliability, heritability

## Abstract

Pupil size and blink rates are heritable but the extent to which they interact with one another has not been properly investigated. Though changes in pupil size due to eye blinks have been reported, they are considered a pupillary artifact. In this study we used the HCP 7T fMRI dataset with resting state eye-tracking data obtained in monozygous and dizygous twins to assess their heritability and their interactions. For this purpose, we characterized the pupil dilation (positive peak) and constriction (negative peak) that followed blink events, which we describe as blink-induced pupillary response (BIPR). We show that the BIPR is highly consistent with a positive dilatory peak (D-peak) around 500ms and a negative constricting peak (C-peak) around 1s. These patterns were reproducible within- and between- subjects across two time points and differed by vigilance state (vigilant versus drowsy). By comparing BIPR between monozygous and dizygous twins we show that BIPR have a heritable component with significant additive genetic (A) and environmental (E) factors dominating the structural equation models, particularly in the time-domain for both D- and C-peaks and amplitude domain for the C-peak. (a^2^ between 42–49%). Blink duration, pupil size and blink rate were also found to be highly heritable (a^2^ up to 62% for pupil size). Our study documents an association between BIPR and wakefulness and indicates that BIPR should not be treated as a coincidental artefact, but part of a larger oculomotor system that we label here as Oculomotor Adaptive System, OAS, that is genetically determined.

## Introduction

Eye-blink rate and pupil size are two physiological measures associated with arousal networks including brainstem dopaminergic, norepinephrinergic, cholinergic and serotoninergic nuclei [1–3]. Both changes in pupil diameter and blink rates have been associated with states of arousal, engagement, and attention [4, 5]. In particular, activation of the Locus Coeruleus (LC) has been associated with changes in pupil dilation [6], which has been shown to covary with the BOLD signal in LC [3], supporting a strong noradrenergic component to its regulation [7]. Additionally other arousal nuclei and their targets have been implicated in pupil diameter. For example, while rapid pupil dilations occurred during phasic adrenergic activity, long-lasting dilatations were associated with tonic cholinergic activity [8]. Weak micro-stimulation of the superior colliculus (SC), a midbrain structure involved in eye movements and attention, evoked transient pupil dilation [9]. These effects are also dependent on arousal states [10]. As for blinks, we recently reported that they correlated with momentary surges in arousal networks as measured with BOLD signals in various brainstem nuclei [11] and have been associated with dopaminergic signaling [1, 12]. Pupil diameter appears to be coupled with alpha EEG activity during inactive (resting) wakefulness [13], whereas blinks have been associated with momentary changes in alpha and delta EEG linking them to states of consciousness [14, 15]. Blink-related EEG activity was also shown to discriminate between different levels of cognitive demand while walking [16].

Eye-blinks and pupil dilations may be initiated by common arousal networks, and blink-pupil synchronization could serve as a biomarker with which to study human vigilance and arousal systems. Interestingly, even though pupil change due to blinks is a known phenomenon [17], this synchrony has been treated as a blink induced pupillary artifact in pupillometry studies [18], or not examined as a possible synchronous/co-incidental phenomenon [19]. On the other hand, both pupil and blink measures appear to be influenced by the level of vigilance. For instance, in a study done in sixty healthy individuals, blink duration was shorter for the alert state compared to the drowsy state [20]. Thus, it is important to analyze pupillary data within different vigilance states.

Increasing evidence suggest that pupil measures are heritable. In a previously study conducted in a large cohort of young Chinese twins (Guangzhou Twin Project; 309 monozygotic (MZ) and 165 dizygotic (DZ) pairs [21]) heritability was found to be approximately 60% for iris thickness and pupil diameter [22]. A study using a large cohort of twin infants (BabyTwins Study Sweden (BATSS), [23]; 510 infant twins assessed at 5 months of age; 281 monozygotic and 229 dizygotic pairs) found that baseline tonic pupil size and pupillary light reflex (PLR) were highly heritable (pupil size, 64% and constriction in response to light, 62%) and linked to genome-wide polygenic risk (PLR) scores for schizophrenia [24]. Another study on 326 female twins (mean age 64 years) from the TwinsUK Adult Twin Registry [25], reported that resting pupil size in complete darkness was strongly heritable with additive genetic effects explaining up to 86% of the variance and environmental factors explaining only 14% of the variability, and for between 31 and 60% of the variability in the PLR [26].

Sympathetic postganglionic neurons project to the dilator pupillae muscle of the iris to produce pupil dilation, while parasympathetic neurons project to the sphincter pupillae muscle to produce constriction [27, 28]. Since blinking and pupillometry are part of the arousal and autonomic nervous system and pupil size and PLR are heritable, it is expected that genetic factors should impact the co-existence of these two physiological responses.

In this study we explored blink rate, blink duration, pupil size, and blink-induced pupillary response (henceforth, BIPR) in the Human Connectome Project (HCP) for the subset collected on monozygotic and dizygotic twins with 7T fMRI scans and that had eye tracking data. Here we show that, while controlling for the arousal state via pupil information, many of the blink and pupil dynamics are reproducible within and across participants, and these dynamics are highly heritable.

## Results

### Eye measures comparing vigilant and drowsy states

**Figure 1:**
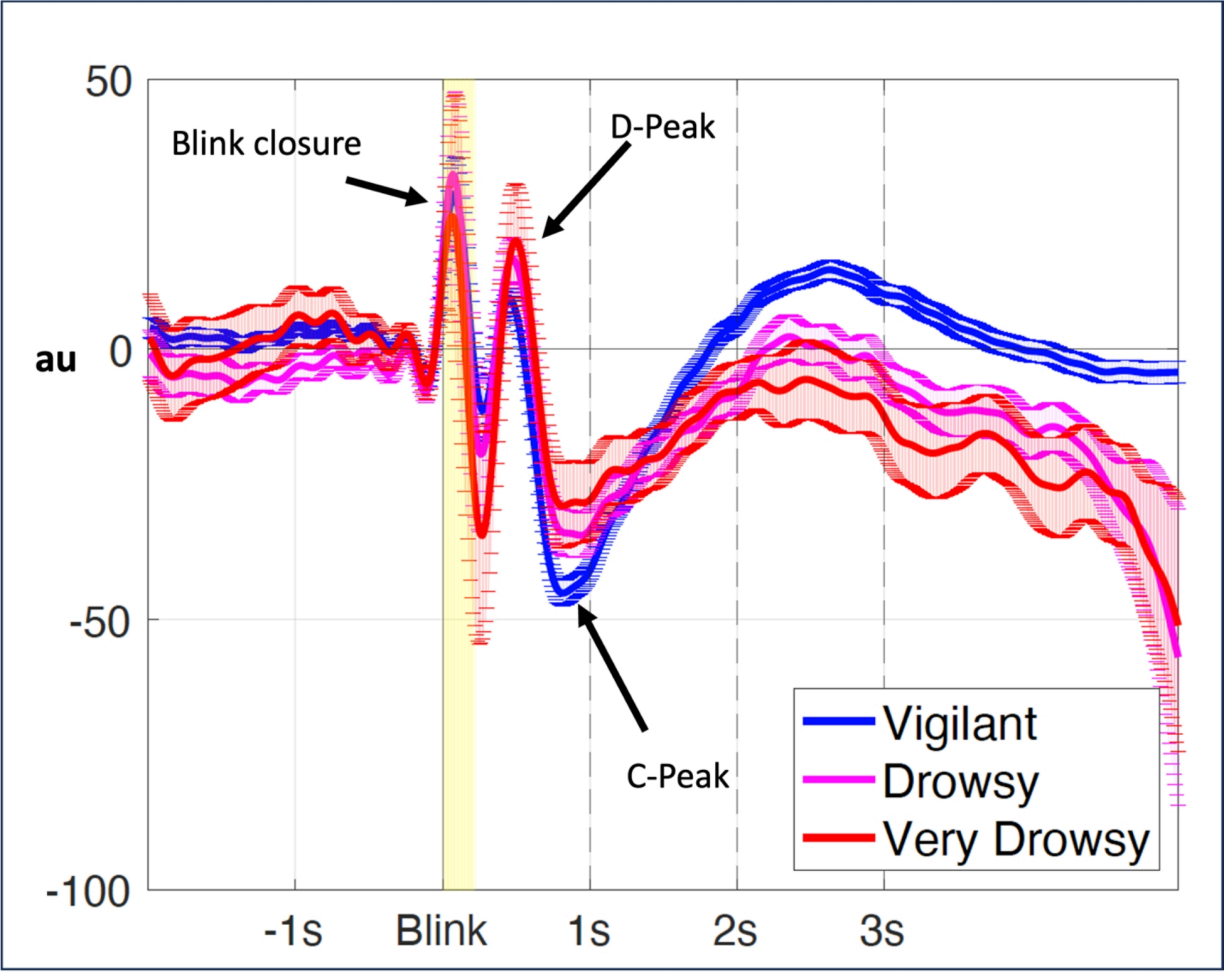
BIPR plots averaged over all runs classified according to the drowsiness state. For this plot 287 vigilant runs, 109 drowsy runs, and 77 very drowsy runs were used.

**Figure 2:**
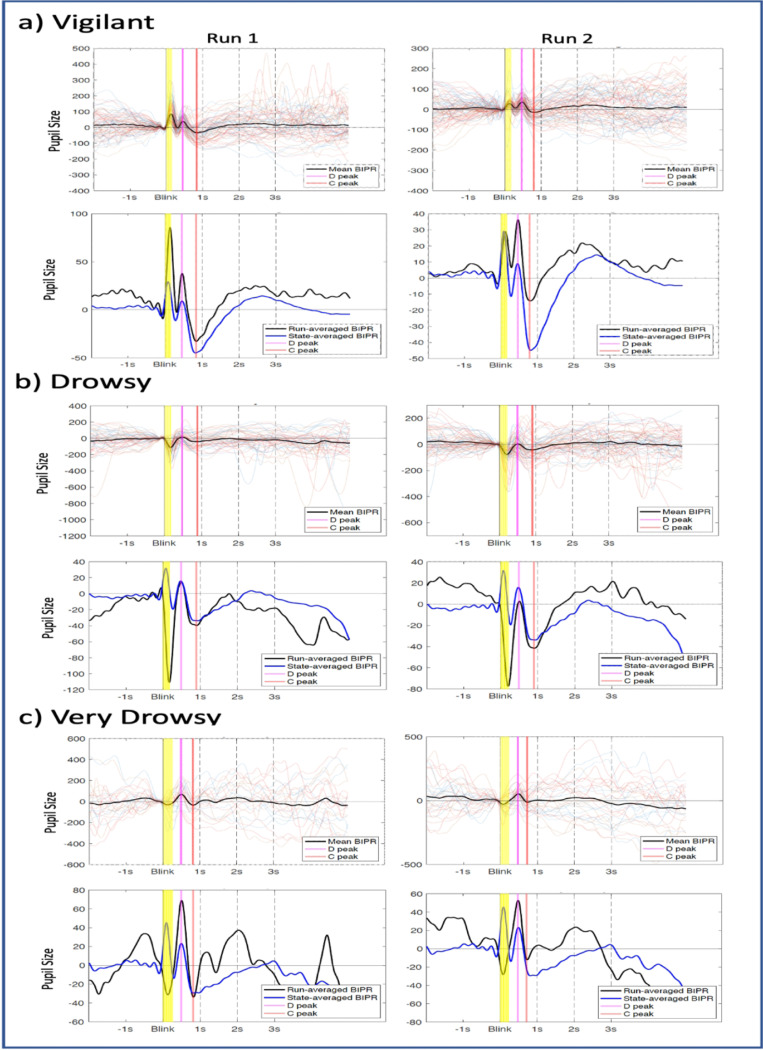
Example BIPR plots for subjects in two runs from a particular state. a) Example subject with two ‘vigilant’ runs, b) another example subject with two ‘drowsy’ runs, and c) two ‘very drowsy’ runs. In the plots (top rows in each a/b/c sections), multi-colored thin lines depict each BIPR epoch collected in that run, and the thick black line is the average BIPR in that run. In the plots in the bottom rows, black line represents the mean BIPR per run while the blue line represents the grand-average (across all runs of all participants) of the same vigilance state. D-peak is marked as magenta, and C-peak is marked as red color line.

The average BIPR across the vigilant, drowsy and very drowsy state are shown in Fig. 1. Six parameters were quantified from the BIPR time series: D-peak time and D-peak amplitude, C-peak time and C-peak amplitude, D-C peak time difference, D - C peakdrop magnitude. Visual inspection of the BIPR time series indicated a positive peak (D), pupil dilation, around 500ms and a negative peak (C), pupil constriction, around 1s. While the ‘amplitude’ and the ‘shape’ characteristics of the BIPR temporal dynamics are partially preserved within a subject in the given vigilant state, and the ‘peak times’ of the positive (D) and negative (C) peaks are highly preserved, these patterns became increasingly variable for the drowsy states as revealed by the examples shown in Fig. 2, with different vigilance states. (We can provide individual peak plots for all the participants in a separate Supplementary Material if requested)

**Figure 3:**
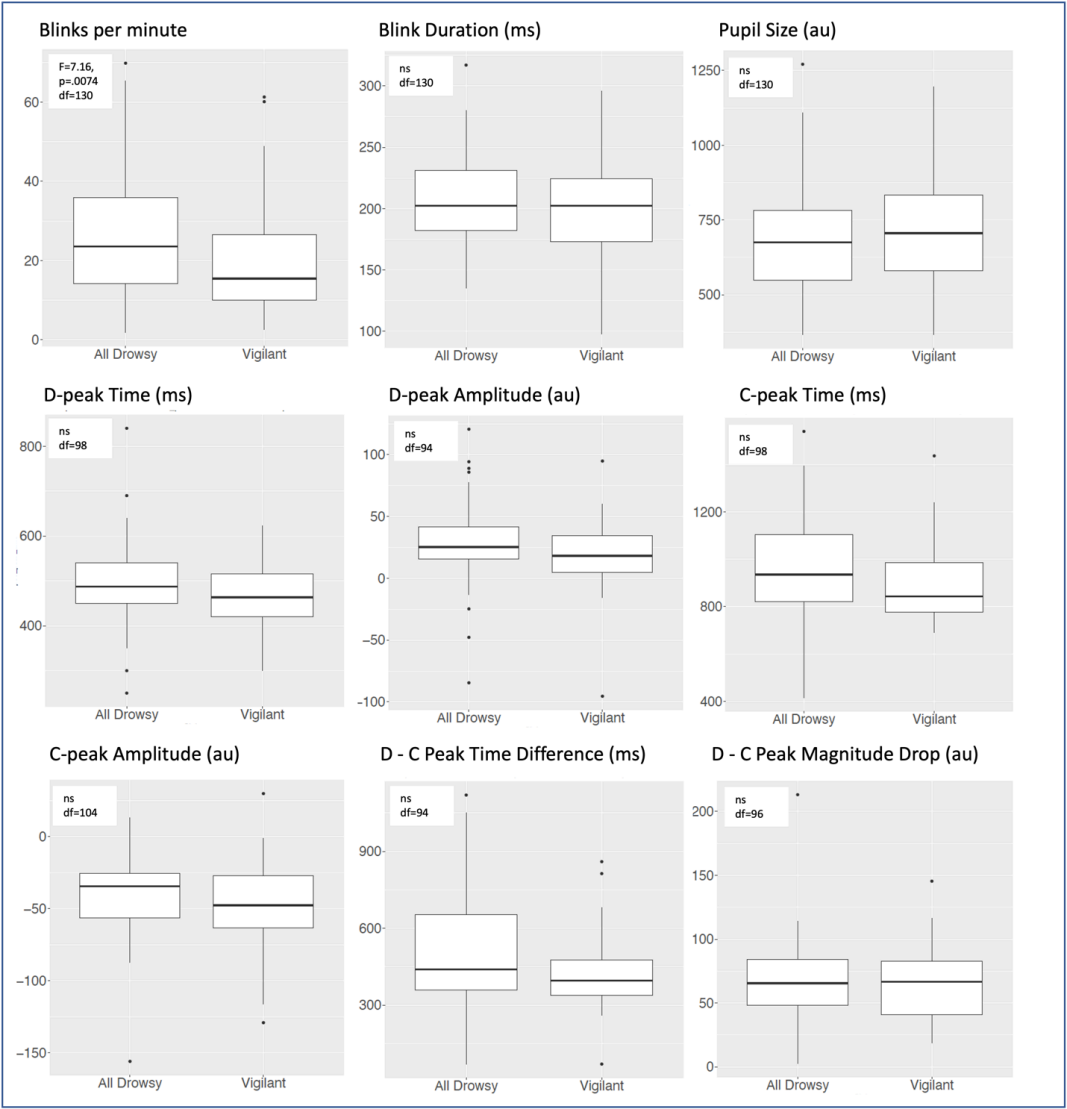
One-way ANOVA analysis of eye measures comparing vigilant and drowsy states. Only subjects with both Vigilant and Drowsy/Very Drowsy runs were used in a pair-wise manner. (Original set runs= 287 Vigilant runs, and 186 All Drowsy runs (109 drowsy runs, and 77 very drowsy runs). Df are shown in the boxes.

We summarize the blink, pupil and BIPR measures and ANOVA results for the vigilant and all drowsy states for participants who had both vigilance states available for a measure shown with degrees of freedom values in Fig. 3 (and see **Supplementary Table 2** for the reporting of mean, standard deviation and coefficient of variation). The outcome of this analysis was that drowsiness slightly increased blink rates (while it slightly decreased pupil size and increased blink durations), but these effects did not survive multiple comparison correction. Similarly, C and D peak times and magnitudes also did not change due to vigilance states, indicating that while vigilance had some influence on the BIPR measures, BIPR features are robust against vigilance, potentially due to genetic determinants and individual differences.

### Reliability

#### Item-reliability

The blink and pupil measures including blink rate, blink duration, and pupil size and the BIPR measures (D, C and D-C peak difference times) and C peak amplitude were highly reliable (Table 1).

### Within-subject reliability

To assess the intra-subject variability, we calculated the correlation coefficient per subject between two runs using normalized (all the numerical measures were centered and scaled) measures. Mean, median, and std of the intra-subject variabilities across subjects were as follows: Vigilant state 0.75 (N = 85, std = 0.25, median = 0.85), and all drowsy states 0.62 (N = 47, std = 0.31, median = 0.73), indicating that BIPR and eye measures for a participant were highly reliable and stable within the vigilance state (see **Supplementary Table 3**).

### Heritability analysis

Table 2 presents intra-pair twin correlations and summarizes the results of genetic model parameter estimates. MZ intra-twin correlations ranged up to 0.62, indicating significant familial influences on the eye measures. For some variables, the MZ correlation was less than twice that of the DZ correlation (r_MZ_ < 2*r_DZ_), suggesting a contribution of shared environmental influences. The overall magnitude of correlations tended to be similar or higher in MZ twins than DZ twins, a pattern consistent with genetic influences.

Next, to test for significant genetic and environmental effects, we fit linear structural equation models to the observed twin data. Based on the pattern of twin correlations (r_MZ_ > 2*r_DZ_), we fit the ADE model for three variables (blink rate, pupil size, and C peak amplitude). In all these cases, the D path could be dropped without a significant decrement in the goodness of fit, and AE was selected as the best-fitting model. For all other variables, the pattern of correlations (r_MZ_ < 2*r_DZ_) suggested the ACE model, where the shared environmental (C) path, but not the additive genetic (A) path, could be dropped without a significant decrement in model fit (indicating that for this phenotype the AE model was the best fitting model, while the CE model could be rejected). The best fitting models were chosen based on Akaike’s Information Criterion (AIC). In summary, the AE model was the best-fitting model for all variables (see also **Supplementary Table 4** for model summaries and comparisons).

Except ‘D-peak amplitude’ (did not reveal reliable fit), and ‘D- C peak magnitude drop’, heritability estimates under the AE model (i.e. the proportion of the total phenotypic variance explained by additive genetic factors A and environmental factors E) showed that variance explained by A (**a^2^**) ranged above .44 to .62, and was significant for blink rate, blink duration, pupil size, D-peak time, C-peak time, and D-C peak time difference. This indicates that basic eye-measures such as pupil size and blink rate and duration, as well as mostly time-dependent BIPR measures are highly heritable with a likely contribution from genetics. On the other hand, D-peak amplitude could not be modelled reliably and D-C peakdrop magnitude was also found to be less-likely to be explained by genetic factors (possibly due to the influence of the D-peak in this measure). However, C-peak amplitude was highly correlated among both MZ and DZ twins, yielding a CE model dominating, emphasizing common genetic and shared factors. (Table 2)

Blink, pupil and BIPR variables used in the analyses are shown in the first column on the left. r_MZ_ and r_DZ_ are intra-pair twin correlations showing the degree of twins’ resemblance with respect to variables shown together with the degrees of freedom (df); a^2^, c^2^, and e^2^ are variance components estimates along with 95% confidence intervals based on the best-fitting model (in ACE; AE or CE would be alternative models that attribute twin resemblance to additive genetic (A), unshared (E) or shared (C) environmental factors. In ADE; E and AE would be the alternative models). Results are presented for the best fitting model only (see **Supplementary Table**).

## Discussion

In this paper we analyzed eye-tracking data collected during the HCP 7T resting state fMRI scans in a group of monozygous and dizygous twins, which allowed to assess the degree of heritability of these measures. In addition, since multiple resting scans were collected, it was possible to estimate the reliability of the blink and pupil measures. We also investigated the interaction between blink and pupil dynamics that we termed the blink-induced pupillary response (BIPR). Though the association between blink and pupil changes had previously been acknowledged it was typically disregarded as an artefact, whereas here we provide evidence that it is a physiological response that is reliable and heritable.

First, we show that while vigilance states had an influence on the measures, when only subjects with paired Vigilant-Drowsy paired runs were used, the effect of vigilance did not reach significance, meaning that genetic factors and individual variability dominated these measures, reducing potential influences from purely vigilance related factors. In another words, variability explained by vigilance is not as strong as the variability explained by the individual and genetic factors.

We also found that within any given state (i.e., vigilant or drowsy) eye measures were very reliable for the vigilant state (> .812), and less so but still strongly reliable for All Drowsy state (> .615). Interestingly, the temporal measures including D and C peak time, and D – C peak time difference were more reliable than the amplitude measures (see **Supplementary Table 2**).

MZ intra-pair correlations were higher than the DZ intra-pair correlations, leading us to test for heritability. We found that AE models (additive and environmental factors) explained the variance better than other models. We found that variance was explained by additive factors (A) from moderate to high degrees (.42 – .62) for all the variables except the D-peak amplitude, and the D – C magnitude drop, where the variance explained by A was small or insignificant, and environmental effects were stronger. Particularly, all the BIPR peak-time measures and C-peak amplitude measure emerged as the most important features related to heritability.

Pupil dilations (and constrictions after blink, BIPR) and blinks are two important but segregated and in most of the times neglected physiological responses that are closely linked to vigilance networks [3, 11]. Our study pointed out the possibility that blink and pupillary consequence after blinking might be driven by similar/overlapping neural mechanisms. For instance in humans, electrophysiological responses (EEG and MEG) to pupil dilations and constrictions are found to be such that pupil dilation peaks are associated with posterior alpha and low beta (8–16Hz) synchrony accompanied/followed by an anterior low (delta) frequency (2–4Hz) desynchronization during wakeful rest [5, 13, 29]. Interestingly in healthy individuals blink related EEG oscillations showed parietal-occipital delta/low-alpha synchrony 500ms after blinking, accompanied by alpha (8–12Hz) desynchronization [3, 30, 31]. Pupil changes were also found to be in synch with the activity of a broad range of ascending arousal nuclei [32]. This is similar to our recent findings showing BOLD activation of ascending brain stem arousal nuclei during blinks [11].

Future work detecting neural activity (i.e., BOLD signal) simultaneously during pupil size changes and blinks where blink-independent phasic pupillary changes could be segregated from the blink-related phasic changes is needed to distinguish the neural correlates underlying these peripheral measures of arousal.

Pupil size is influenced both by the sympathetic nervous system through noradrenergic connections via the Suprachiasmatic Nucleus (SCN) and the LC acting over the dilator muscle, which dilates the pupil, and by the parasympathetic system over the Edinger-Westphal nucleus (EW) through cholinergic pathways acting on the sphincter muscle, which constricts the pupil [28]. LC also has inhibitory effects over the EW. If the cholinergic antagonist Tropicamide is used to block ACh on the sphincter muscles, the phasic pupil dilation is diminished and the tonic pupil dilation is delayed [33, 34], while Phenylephrine, an adrenergic a-1 receptor agonist preserved phasic pupil dilation and partially preserved the tonic pupil size [33], indicating that the iris sphincter muscle and the parasympathetic system play a primary role not only in constricting the pupil but also in controlling rapid pupil dilation. Sustaining pupil size on the other hand is the act of both the dilator and sphincter muscles via both cholinergic and noradrenergic systems. Blinks emerge in a very fast manner and the light hitting the retina changes within tens of milliseconds during which arousal systems might find a moment to reset their activity. Blinking can allocate sympathetic and parasympathetic systems balancing the neural synchrony in subcortical and cortical brain regions, in an individual specific and heritable manner.

Lastly, the orientation response [18] may not only be a light reflex, but also part of a system most likely mediated by brainstem nuclei including Superior Colliculus (SC) and Locus Coeruleus (LC), leading to the orienting of covert and overt visual attention, to help maintain arousal levels at satisfactory levels. As mentioned in studies by [9, 35] and emphasized in [36], orienting responses could be externally driven or as in our case, internally driven, such that internal arousal surges initiated by the ascending arousal nuclei generating (or co-incident with) the spontaneous eye-blinks [11] could be part of a system that can be named as the ‘Oculomotor Adaptive System’, OAS, that includes the SC. For instance, the initial pupil dilation after the SC stimulation in Bell et al. study (driven by the sympathetic system) and its timing looks very similar to the D-peak BIPR component. It would be valuable for future studies to provide further neurophysiological evidence about this system and to investigate it across various neuropsychiatric conditions to determine its potential as a biomarker for diagnosis and for predicting or monitoring treatment responses.

### Potential constraints

#### Unit of measure

As units of measure we used the arbitrary units given by EyeLink1000 as reported in the HCP database. Calibration was conducted and the distance measures between the eyes and the camera were registered in the data acquisition computer as default settings by the HCP staff before each experiment reaching good standards.

#### Head motion and pupil detection

Concerns could be raised about the effects of head movement on pupillary responses. However, because head movements introduce significant artifacts in the MRI measures the experiments require that the head of the participants while lying in the magnet be strictly stabilized, which is achieved by the placement of foams around the head and inside the head coil (something like a helmet). Thus, we are confident that the head movement was minimal in the 7-T MRI setup. We also show that frame-wise displacement (FD) was not significantly different between vigilance status. For the HCP data set the EyeLink did not record the eye video so instead we monitored eye closures when we detected a pupil loss (marked as ‘0.0’ in the file). We detected a few weird cases in the HCP eye tracking raw data (.asc file) such that any ‘0.0’ valued intervals (missing pupil timepoints) that were regarded and labelled as ‘EBLINK’ even though they could be as short as 10ms and as long as 30 seconds or more. That’s why in our analysis we did not rely on their labelling. Instead, we went through the raw data and selected blinks when the pupil value was 0.0 and continued anywhere from 40ms to 400ms. Thus, the eyeblink onset time was selected as the moment when the pupil value turned to 0.0 and stayed zero within this window range.

For pupil detection under partial eyelid, the EyeLink system mainly uses the ellipse-fitting pupil model. This is preferable if the pupil is significantly occluded (for example by the eyelids) as the ellipse fitting algorithm may give a more accurate estimation of pupil position. The ellipse-fitting mode decreases drift potential and copes well with pupil occlusion but at the cost of a higher noise level. Please see https://fchetail.ulb.ac.be/wp-content/uploads/EyeLink-1000-User-Manual-1.5.0.pdf, section ‘Pupil Tracking Algorithm’. We think that partial eyelid closures that are strong enough to block pupil detection even with the elliptical approach under heavy drowsiness status may take much longer than 400ms, leading to micro sleep episodes. In the Supplementary material we present example figures showing original FD (circle represent a value per TR/Volume). Note that movements are extremely small, most probably respiration related head motions. Another figure is also presented with the FDs overlaid on Fig. 1 in the Supplementary Material. Another plot shows the distribution of the correlation values between pupil size and the FD across runs to see whether FD and pupil size are distributed normally (Supplementary Material). We are confident that head motion was not a problem and show with these results that the blinking and head motion measures are independent components.

Lastly, sometimes individual differences in the pupil-cornea color contrast were weak not letting the system detect the corneal reflection and the pupil, and also the use of contact lenses can impact pupil detection and size measure.

### Microsleeps

There can be ‘microsleep’ episodes particularly during the drowsy scans. Generally, microsleeps are regarded as eye-closures of more than 4s [37] and initiation of microsleeps can induce wide negative BOLD signals in fMRI, and reduce respiration and heart rate both of which recover back to the initial values as the micro-sleep ends. However, the most reliable measure of detecting sleep is the EEG (Electroencephalography) using alpha, theta and low delta frequency oscillations, detection of K-complexes and spindles, which are sleep stage dependent and part of up- and down-states in sleep. In this dataset there were no EEG recordings. These vigilance fluctuations (and drowsy context) can induce dynamic effects on the pupil size and consecutively on BIPR, which is consistent with the results we show in this manuscript.

## Conclusion

In conclusion our results indicates that the pupil diameter changes that follow blinks (BIPR) are reliable, and provide strong evidence that they are heritable. Individual variability and genetic factors dominate and influence the potential effect of drowsiness, thus future studies exploring the effects of vigilance on pupillary measure should aim to control for inter individual variability. Future work is needed to validate the BIPR measure as a biomarker of arousal and to investigate further the neurobiological signals underlying it.

## Materials and Methods

### 7T HCP dataset

HCP 7T dataset contains data from 184 participants (48 MZ, and 42 DZ twins and 4 individuals who were the siblings from the twin families) who underwent up to four resting state fMRI runs done over two separate days -two runs in each day interspersed with task scans [38]. HCP dataset has been extensively used in the recent years to examine genetic [39] and non-genetic brain function [40]. Participants were positioned supine in the MR scanner and during the resting state scan stared at a cross on the center of the screen. In total, we found that 177 subjects had four runs of resting state fMRI scans, two had three runs, four had two and one participant had only one run in the database. Among these runs some lacked eye tracking data leading to 131 participants with four runs of resting state and eye tracking data, 14 participants with 3 runs, one with two runs and two participants with one run (leading to 570 runs across 148 participants). Of these, 10 runs had to be excluded due to problems in the eye-tracking file either not being recorded properly, missing data points, or showing problems in opening/importing files into the computer software. Final set comprised 560 runs across 148 participants (39 MZ, and 32 DZ twins, and 6 individuals (who were either the siblings of the initial twin families, or the participants who lost their twin partner from the original set because no usable resting state runs were available from their twin partners). Further categorization of the eye-tracking data is explained below.

### Characteristics of participants included in the study

Participants initially consisted of 39 monozygous, 32 dizygous twins and 6 non-twins. Their demographic characteristics are shown in **Supplemental Table 1**. These groups did not differ in age, race, or sex. In the heritability analysis we only used the data from the twins.

### Eye-tracking

Eye-tracking was conducted with the Eye Link 1000 system with 1kHz sampling rate (some runs were with 500Hz) from the right eye. We conducted the following pre-processing steps: i) used R package ‘eyelinker’ (https://cran.r-project.org/web/packages/eyelinker/vignettes/basics.html) to extract all runs with usable continuous eye-tracking data, ii) followed by MATLAB routines, runs with 500Hz sampling rate were up-sampled to 1kHz, iii) time period between 40–400ms of pupil loss was marked as eye-blinks (blink onset moment was the starting time of the pupil loss), iv) missing data points due to eye-blinks were cubic-spline interpolated with the neighboring points (https://github.com/jacobaparker/PRET/blob/master/blinkinterp.m), v) pupil size time series were then filtered with forward-backward FIR filter with values: low cutoff .02Hz, high cutoff 4Hz, vi) data was then downsampled to 100Hz.

### Final selection of runs and participants

Each resting scan/run was classified into one of four states: a) vigilant (when less than 10% time of the run the pupil was not detected); b) drowsy (10%−40% pupil loss in a run); c) very drowsy (40%−75% pupil loss in a run); and d) discarded (more 75% pupil data loss). (**Supplementary Figs. 1 and 2**)

**Figure 4:**
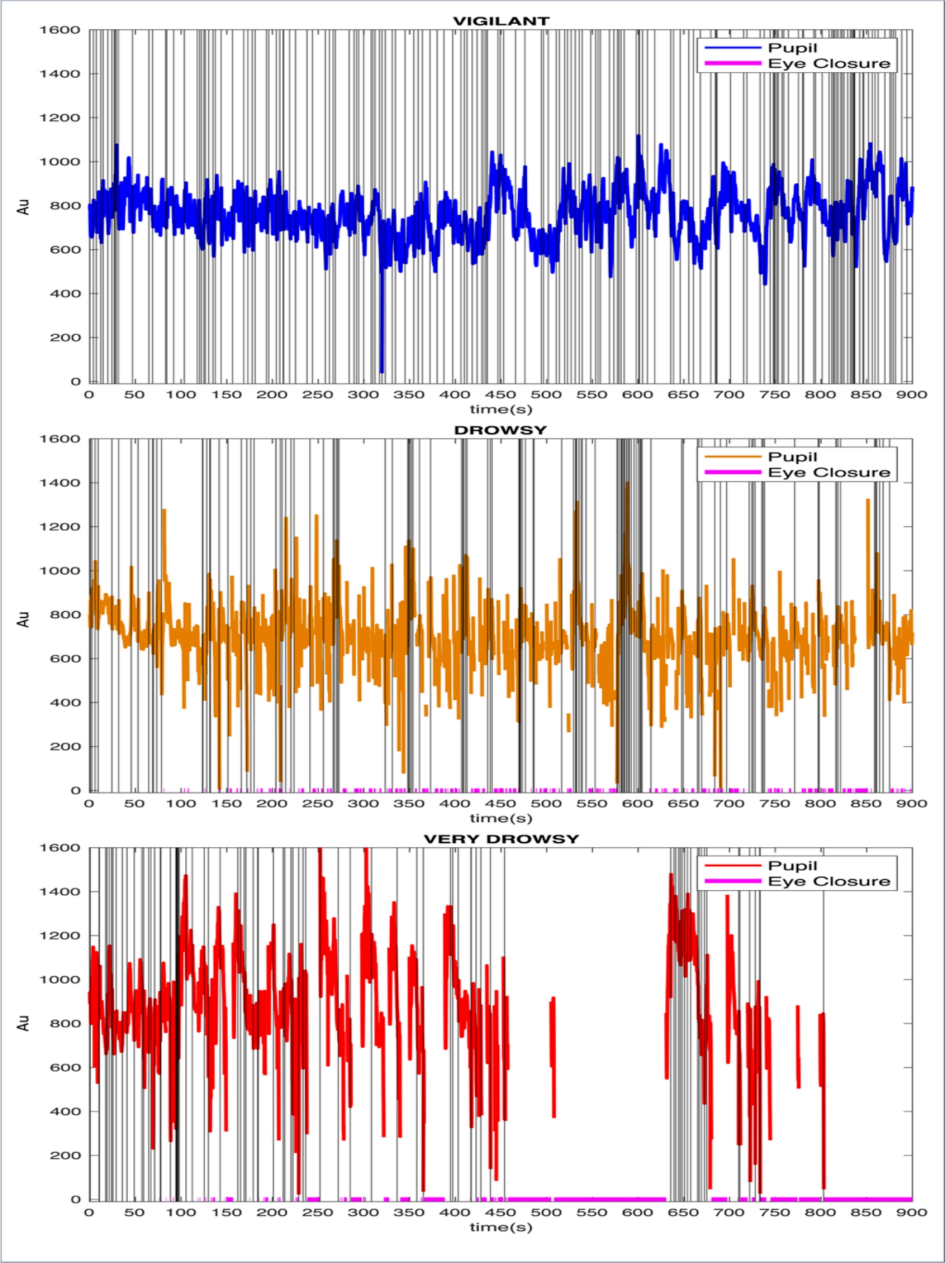
State classification and examples. Blink moments marked with gray vertical lines and the eye closures marked with magenta color small vertical lines at the bottom of the figures. We also set the y-axis limits to be consistent across the vigilance states.

Of the total of 560 runs from the 7T HCP dataset 291 were classified as vigilant runs (52% of the total runs across 111 subjects), 111 as drowsy runs (19.8% of the total runs across 75 subjects), 85 as very drowsy runs (15.2% of the total runs across 59 subjects) and 73 runs were discarded (13% of the total runs across 41 subjects). 58 subjects had both vigilant and drowsy runs available; 32 subjects had both vigilant and very drowsy runs, and 68 subjects had vigilant and any type of drowsy runs. After exclusion of the ‘discarded’ runs, a total of 487 runs across 141 participants remained. We combined drowsy and very drowsy runs, which we labelled as “All Drowsy” and used to compare drowsy states against vigilant states. In the final analysis reported here, total number of subjects for the Vigilant state was 85 (where 24 of them provided 2 runs, 30 of them provided 3 runs and 32 of them provided 4 runs possessing vigilant state; total of 266 runs), and total number of subjects for the All Drowsy state was 47 (where 32 of them provided 2 runs, 11 of them provided 3 runs and 3 of them provided 4 runs possessing either Drowsy or Very Drowsy states which were merged as All Drowsy). Pupil time-series along with eye-closures and blinks from 3 different vigilance states samples are shown in Fig. 4.

### Additional drowsiness measures

We computed head motion during a scan with the framewise displacement (FD) measure, which showed that head motion was similar across vigilance states (**Supplementary Fig. 1; see Suppl. Figure 3** for FD and pupil size together in a time-series manner). We also report how drowsiness changed in time across subjects; showing that the longer the time participants stayed in the scanner the more likely they were to close their eyes at the later sections of a run (**Supplementary Fig. 2**).

### Extraction of pupil size, blink rate, blink duration and blink-induced pupillary response (BIPR)

After preprocessing and classification of the pupil time series across runs and subjects, we calculated blink rate (average blinks per minute of total duration while eyes open, excluding eyes-closed times), blink duration, average pupil size, and blink-induced pupillary response (BIPR) measures for each vigilance state and subject. BIPR represents the pupil size changes onset to the eye-blink. For each blink moment, we extracted pupil size changes from 2s before the blink to 3s after the blink, baselined the pupil time series to the mean value in the − 200ms to 0ms interval, which we describe as BIPR epoch, and used it to calculate grand mean BIPR of each run. An epoch where more than 40% of the data points within the 0s – 3s interval were missing/unavailable were excluded, because for a few blink epochs the pupil data may not be available for a long duration of time due to longer eye closure after blinks. We then averaged all the available BIPR epochs per run per subject, yielding an average BIPR time series for the run. In any dependent measure, outlier values above or below two inter-quartile range from the 25% and 75% percentiles (respectively) were excluded from the final analysis.

### State dependent changes in the blink, pupil and BIPR measures

We conducted one-way ANOVA comparing ‘vigilant’ and ‘all drowsy’ states across nine measures of interest (six BIPR measures, blink rate, blink duration and pupil size). Since blink measures are assumed to be heritable and there are individual differences with high test-retest reliability (see below), in this analysis we only used subjects with both a Vigilant and All Drowsy state available, creating a paired dataset for ANOVA analysis (i.e., reducing the influence of the subjects with un-paired data over the vigilance states on statistics). We report multiple comparison corrected (adjusted alpha = .05/9 = .0056) results.

### Test-retest reliability for variables (item-reliability) and for subjects (intra-subject reliability)

Total number of subjects for the Vigilant state was 85 (where 24 of them provided 2 runs, 30 of them provided 3 runs and 32 of them provided 4 runs possessing vigilant state; total of 266 runs). Total number of subjects for the All Drowsy state was 47 (where 32 of them provided 2 runs, 11 of them provided 3 runs and 3 of them provided 4 runs possessing either Drowsy or Very Drowsy states which were merged as All Drowsy). We revised our analysis with the method defined by [41] using theICC2,k approach and a linear model with 2-way random effects (subjects and runs) implemented in R software as the ‘SimplyAgree’ package described as ‘reli_stats’ function.

Note that, if a subject had two runs from one state (i.e., vigilant) and two from another state (i.e., drowsy), these observation-pairs were included as two separate pairs in calculations for each state. For all the analysis mentioned above, all the available runs and subjects were used and pooled together without considering their twin status.

### Intra-pair twin correlations and genetic heritability analysis

To estimate the relative contribution of genetic and environmental sources to the total phenotypic variance, we fit linear structural equation models using the OpenMx package [42, 43] in R software [44]. We previously showed heritability of brain EEG signals in error monitoring for twins with Mx model [45]. These models assume that phenotypic variance arises from: additive genetic influences (A), non-additive genetic influences (D) [including within-locus allelic interaction (dominance) and between-locus interaction (epistasis)], environmental influences shared by family members (C), and individually unique (unshared) environmental influences (E) [46]. The non-additive component (D) is the additional variance resulting from the deviation from the additive effects. It can only be present if additive effects are present. Importantly, A, D, and C increase, while E decreases, intra-pair twin similarity. When using data from twin pairs reared together, it is only possible to test three of these four components simultaneously, and a decision regarding whether to test an ADE or an ACE model is made based upon the observed twin correlations [47]. To decide which model to use, first we calculated twin intra-pair correlations for MZ and DZ twins for each variable. Specifically, if the MZ correlation is equal to twice the DZ correlation, then AE is the most parsimonious and the best fitting model. If the MZ correlation is smaller than twice the DZ correlation this indicates the possibility of shared environmental effects, warranting consideration of ACE and CE models. On the other hand, if the MZ correlation is greater than twice the DZ correlation, then the contribution of non-additive genetic effects is possible, and the ADE model should be considered because a DE model is biologically implausible. The fit of nested sub-models was tested by dropping individual paths from the full model, with the significance of individual paths tested by comparing the fit of the restricted sub-model with the fit of the more general model using a χ2 test with degrees of freedom corresponding to the difference in the degrees of freedom between two models. If dropping a path significantly reduced the goodness of fit, the path was retained in the model, otherwise the more parsimonious model was chosen (i.e. the one that accounted for the variance equally well, but with a fewer number of parameters). To choose between non-nested models (AE and CE in case of ACE model), we used Akaike’s Information Criterion (AIC), where AIC = χ2 − 2df. Lower AIC values indicate better fit. Path coefficients for the best-fitting models were estimated using the method of maximum likelihood, and the goodness of model fit was indicated by − 2 times the log likelihood (− 2LL). Heritability was estimated as the percentage of the total variance of the trait attributable to genetic factors. In this analysis, we used only MZ and DZ twins, and put all the twin measures (data pairs) across vigilance states together per each twin type.

## Figures and Tables

**Table 1:** Test-retest reliability (Intraclass Correlation Coefficient, ICC) of each variable for Vigilant and All Drowsy runs across subjects (All Drowsy runs include Drowsy and Very Drowsy runs). Total number of subjects for Vigilant state was 85 (where 24 of them provided 2 runs, 30 of them provided 3 runs and 32 of them provided 4 runs possessing vigilant state; total of 266 runs). Total number of subjects for All Drowsy state was 47 (where 32 of them provided 2 runs, 11 of them provided 3 runs and 3 of them provided 4 runs possessing either Drowsy or Very Drowsy states which were merged as All Drowsy). ICC2k model (two-way random effects model with average agreement across trials) Total Reliability above 0.5 was shown in bold font.

Variable	Vigilant (N = 86)	All Drowsy (N = 47)
Blinks per minute	**.942**	**.872**
Blink duration	**.922**	**.919**
Pupil size	**.940**	**.834**
D peak time	**.886**	**.874**
D peak amplitude	**.812**	**.750**
C peak time	**.900**	**.903**
C peak amplitude	**.841**	**.642**
D - C peak time dif.	**.842**	**.615**
D – C peak mag. drop	**.883**	**.687**

**Table 2.** Intra-pair twin correlations of the MZ and DZ twin pairs and heritability analysis

Variable	r_MZ_ (N = 44 twin-pairs)	r_DZ_ (N = 36 twin-pairs)	Best Fitting Model	a^2^ (95% CI)	c^2^ (95% CI)	e^2^ (95% CI)
Blinks per minute	.535*** (df = 40)	.221 (df = 31)	AE	.49 (.23–.68)		.50 (.32–.76)
Blink duration	.523*** (df = 40)	.235 (df = 31)	AE	.44 (.18–.63)		.55 (.36–.81)
Pupil size	.625*** (df = 41)	.506** (df = 32)	AE	.62 (.43–.74)		.37 (.25–.56)
D-peak time	.56* (df = 17)	.61* (df = 10)	AE	.49 (−.08–.72)		.51 (.28–.91)
D-peak amplitude	− .23 (df = 16)	− .081 (df = 8)	-		-	-
C-peak time	.54* (df = 14)	.082 (df = 15)	AE	.42 (−.01–.69)		.57 (.49–.98)
C-peak amplitude	.51* (df = 20)	.54* (df = 14)	CE		.49 (.23–.68)	.50 (.31–.76)
D - C peak time difference	.59* (df = 13)	− .24 (df = 11)	AE	.44.1 (.27–.51)		.56 (.28–.97)
D - C peakdrop magnitude	.02 (df = 17)	−41 (df = 9)	E			.94 (.55–1.37)

* Significance of twin correlations: p < 0.05.

** Significance of twin correlations: p < 0.01 (one-sided).

*** Significance of twin correlations: p < 0.001 (one-sided)

## Data Availability

Human dataset used in this study is publicly available from the HCP1200 database where data can be downloaded via the Connectome DB software (https://www.humanconnectome.org/study/hcp-young-adult/article/reprocessed-7t-fmri-data-released-other-updates). Genetics information should be accessed via a granted permission by the HCP committee which is reported as “Restricted.xls” (contact Jennifer Elam, Ph.D., Scientific Outreach, Human Connectome Project; elam@wustl.edu). Rest of the data processing scripts and summary data produced by the authors will be available at https://github.com/demiralsb/Blink-Arousal upon the receipt of this manuscript.
